# Leptin and leptin receptor expression as biomarkers for breast cancer: a retrospective study

**DOI:** 10.1186/s12885-023-10617-8

**Published:** 2023-03-20

**Authors:** Yan Wang, Lili Du, Jiexian Jing, Xianwen Zhao, Xing Wang, Shenghuai Hou

**Affiliations:** 1 Department of Etiology and Tumor Markers Laboratory, Shanxi Province Cancer Hospital/ Shanxi Hospital Affiliated to Cancer Hospital, Chinese Academy of Medical Sciences, Taiyuan, Shanxi China; 2 Department of Colorectal and Anal Surgery, Shanxi Province Cancer Hospital/ Shanxi Hospital Affiliated to Cancer Hospital, Chinese Academy of Medical Sciences, No. 3 Zhigongxin Street, Xinhualing District, Taiyuan, 030013 Shanxi China

**Keywords:** Breast cancer, Leptin, Leptin receptor, Survival

## Abstract

**Background:**

Effective screening and treatment have reduced the number of women dying from breast cancer (BC). However, the long-term sequelae of BC treatment and psychosocial factors seriously affect the life quality of BC patients and survivors. Therefore, the discovery and application of targeted biomarkers to improve the functional outcome and life quality of BC patients is necessary.

**Aims:**

To explore the impact of leptin (LEP)/ leptin receptor (LEPR) expression on occurrence and survival of BC.

**Methods:**

Totally 132 primary BC and 66 non-BC patients who underwent surgery in department of breast surgery in Shanxi Cancer Hospital from January to October in 2009 were enrolled in this retrospective study. LEP and LEPR were examined in BC tissues, benign breast tissues, para-carcinoma tissues using immunohistochemical staining. Kaplan–Meier curve was generated to test survival time.

**Results:**

The high level expression of LEP and LEPR in BC tissues were significantly higher than that in benign breast tissues and in para-carcinoma tissues (all *P* < 0.05). The LEP expression in patients with lymph node metastases was significantly higher than that in patients without lymph nodes metastases (*P* = 0.002). LEPR expression was correlated with higher Ki-67 rate (*P* = 0.002). LEP and LEPR both had no impact on survival (all *P* > 0.05).

**Conclusions:**

High LEP/LEPR expression were risk factors for occurrence of BC, but without impact on survival.

## Introduction

Approximately more than 2 million women worldwide are diagnosed with breast cancer (BC) each year. The number of women dying from BC has fallen thanks to screening and effective treatment. However, radical mastectomy, chemoradiotherapy, and targeted therapy all have different degrees of harm to BC patients, leading to long-term sequelae of BC treatment. For BC patients and survivors, long-term sequelae as well as many psychosocial determinants, such as health care system factors, work constraints, spirit, and coping, seriously affect the life quality of BC patients and survivors [1, 2]. BC is arguably responsible for global burden and disability. Immunometabolism is found to be a new approach for tumor immunotherapy. Drugs to control dyslipidemia can well control lipid metabolism, thereby improving the tumor immune microenvironment of BC patients and inhibiting the recurrence and metastasis of BC [3]. It can be argued that health care today is not only about prolonging the life of patients, but also about maintaining and improving their health. Therefore, the discovery and application of targeted biomarkers to improve the functional outcome and quality of life of breast cancer patients is necessary. Obesity has been proved to be an independent risk factor for development of (BC, which was induced by adipose tissue secreted peptides [4–6]. As a neuroendocrine hormone exclusively generated by adipose tissue, Leptin (LEP) plays multiple biological functions, such as regulating insulin secretion and promoting cell proliferation and angiogenesis, through receptor-mediated pathways [4]. The relationship between LEP and cancer progression, has been widely explored [7–11].

It has been confirmed that LEP can significantly promote proliferation of BC cells compared with that of normal breast cells [12], and only BC cells will respond to LEP rather than normal breast cells [13]. Our recent study also found that BC patients had higher LEP levels than healthy controls [14, 15]. LEP plays biological functions by binding to leptin receptor (LEPR). LEPR is a single-pass transmembrane protein which plays a regulatory role in metabolism. Metabolic disorder is a direct cause for obesity and obesity-related diseases [16–18]. Previous studies showed that LEPR and LEP genes were two important genes related to obesity, and also closely related to BC [19, 20].

Present study aims to detect the expression of LEP and LEPR in BC patients, and further explore the impact of LEP/LEPR on occurrence and survival of BC.

## Materials and methods

### Specimen

**Fig. 1:**
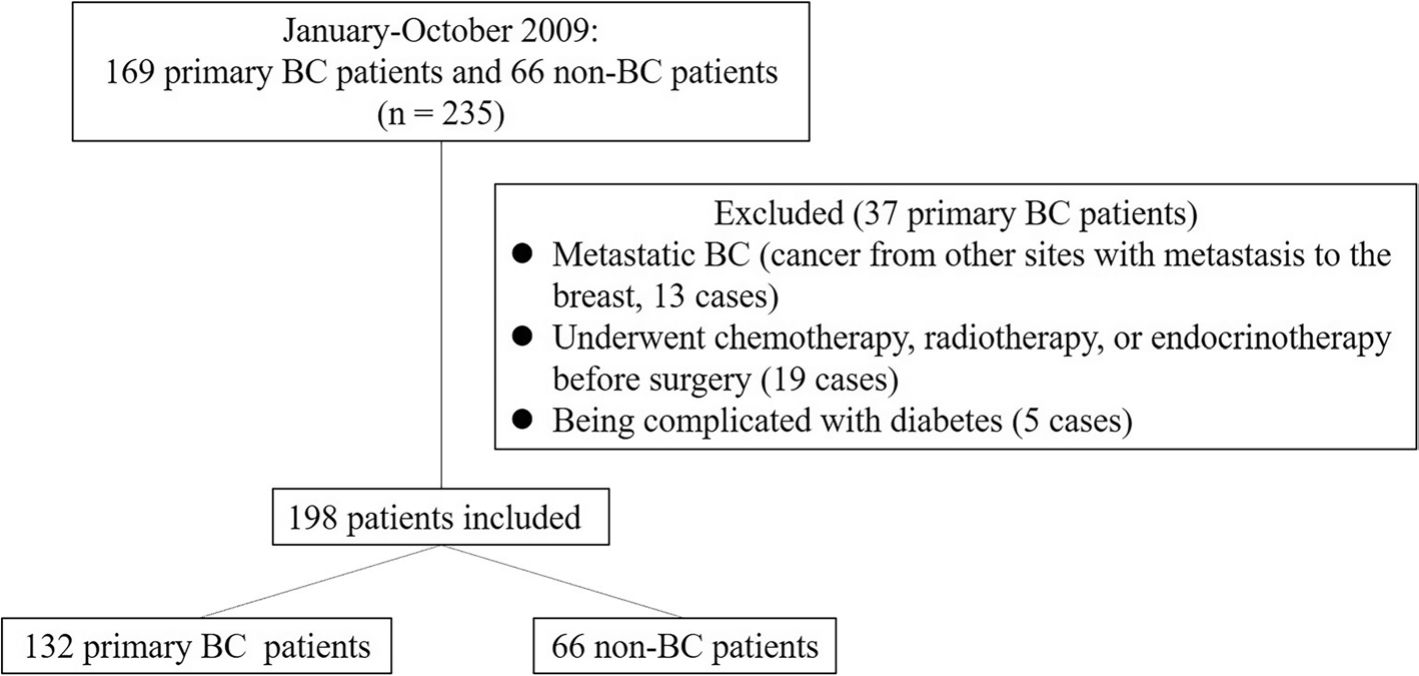
The flowchart of patient enrollment. A total of 198 BC patients and 66 non-BC were primarily enrolled. Thirteen cases were excluded for metastatic BC, 19 cases were excluded for being performed chemotherapy, radiotherapy, or endocrinotherapy before surgery, and 5 cases were excluded for being complicated with diabetes. Totally 132 BC patients and 66 non-BC patients were finally enrolled

Totally 132 primary BC and 66 non-BC patients who underwent surgery in department of breast surgery in Shanxi Cancer Hospital from January to October in 2009 were enrolled in this retrospective study. Patients meeting these criteria were included: (I) Female patients with breast cancer or benign breast disease; (II) Age between 30 to 70 years; (III) No Chemotherapy, radiotherapy and endocrinotherapy before surgery; (IV) pathologically confirmed after surgery. Patients with diabetes or malignancies other than breast cancer were excluded from analysis. The flow chart was shown in Fig. 1. All specimens collected during surgery were fixed with 10% neutral formalin within 4 h after surgery and embedded in paraffin. Age, menopausal status, height, weight and circumference of waist and hip were collected and used to calculate body mass index (BMI) and ratio of waist to hip (WHR).

All procedures performed in this study involving human participants were performed in accordance with the ethical standards of Shanxi Medical University Committee and with the 1964 Helsinki declaration and its later amendments (approve number: 2011134). 

### Reagents and instruments

Rabbit LEP (A-20) and Rabbit LEPR (M-18) antibody to human were purchased from Santa Cruz, USA. Rapid immunohistochemistry EnVision™ Kit 5004, and diaminobenzidine (DAB) were purchased from Fuzhou Maixin Biotech, China.

### Immunohistochemistry

BC and breast fibroma tissues were prepared as tissue microarray samples using a tissue microarray preparation machine (Hengtai Co, LTD, Liaoning), and then sliced into 4 um serial sections. After incubation at 60°C for 4 h, the sections were immediately dewaxed, hydrated, and hot repaired under high pressure. After blocking endogenous peroxidase activity using H_2_O_2,_ the sections were incubated with a drop of LEP/LEPR antibody at 4°C for overnight (LEP: 1:100, LEPR: 1:90), immersed with EnVision™ solution, stained with DAB and counter-stained with hematoxylin. After dehydration, transparent sections were mounted and examined with microscope. Human adipocyte tissues were used as positive controls, and samples without incubation of LEP or LEPR antibodies were used as negative controls.

### Result evaluation

LEP is expressed on cytosol and LEPR is expressed on both cytosol and membrane. Yellow or yellowish brown staining was considered as positive. The percentage of positive cells under optical microscope was calculated. Samples with less than 10% positive cell were given 1 point, 2 points when 10–50% cells were positive, and 3 points when ≥ 50% cells were positive. Signal intensity was scored as weak (1 point), moderate (2 points), or strong (3 points). Both scores were multiplied [21], and the resulting score was used to categorize LEP/LEPR expression as low (< 4) or high (≥ 4). Estrogen receptor (ER), progesterone receptor (PR) and Ki-67 are expressed in the nuclei of cancer cells. Yellowish brown particles were considered as positive.

### Statistical analyses

All the data collected in this study were analyzed using SPSS 22.0 software. Normally distributed measurement data were expressed as mean ± standard deviation (SD), and the comparisons were examined by Student-t test and Mann–Whitney test (non parametric distribution). Categorical data were expressed as n (%), and the differences between the two groups were examined by chi-square analysis or Fisher's exact test. The correlation between LEP/LEPR expression and clinical characteristics were analyzed by chi-square analysis. Survival time was calculated from surgery to death of patient or the last follow-up time. Kaplan–Meier methods was used for survival analysis. *P* < 0.05 was considered statistically significant.

## Results

### General information

Of these 132 patients, 10 patients were with well-differentiated, 60 patients were with moderately differentiated, and 62 patients were with poorly differentiated BC. Median age of these patients was 46 (30–70) years old. Sixty-six patients with benign breast diseases and 30 para-carcinoma tissues, which were normal breast tissues and 4 cm away from the tumor tissues from the 132 cancer patients, were included in controls. Among the patients with benign breast diseases, 37 had fibroadenoma, 15 had multiple intraductal papilloma and 14 had lobular proliferative diseases. They were aged between 34 and 68 years old with median age of 43 years old.

There was no difference of BMI (24.04 vs 23.28 kg/m^2^, *P* = 0.142) and WHR (0.86 vs 0.82, *P* = 0.241) between BC patients and benign ones. Baseline characteristics of patients with breast cancer were shown in Table 1.

**Table 1:** Baseline characteristics of patients

**Characteristics**	Number of patients
**Patients with breast cancer**	132
**Age (years)**	
≤ 55	110
> 55	22
**BMI [**kg/m^2^,**(**$\overline{\upchi } \pm s$)]	24.04 ± 3.47
**WHR (**$\overline{\upchi } \pm s$)	0.86 ± 0.32
**Menstrual status**	
Premenopausal	98
Postmenopausal	34
**Histological type**	
Invasive ductal carcinoma	58
In situ ductal carcinoma	55
Invasive lobular carcinoma	9
Lobular neoplasia	10
**TNM stage**	
0	2
I	37
II	71
III	20
IV	2
**Status at last follow-up**	
Alive`	114
Dead	7

*BMI* body mass index, *WHR* waist-to-hip ratio

### LEP and LEPR expression

**Fig. 2:**
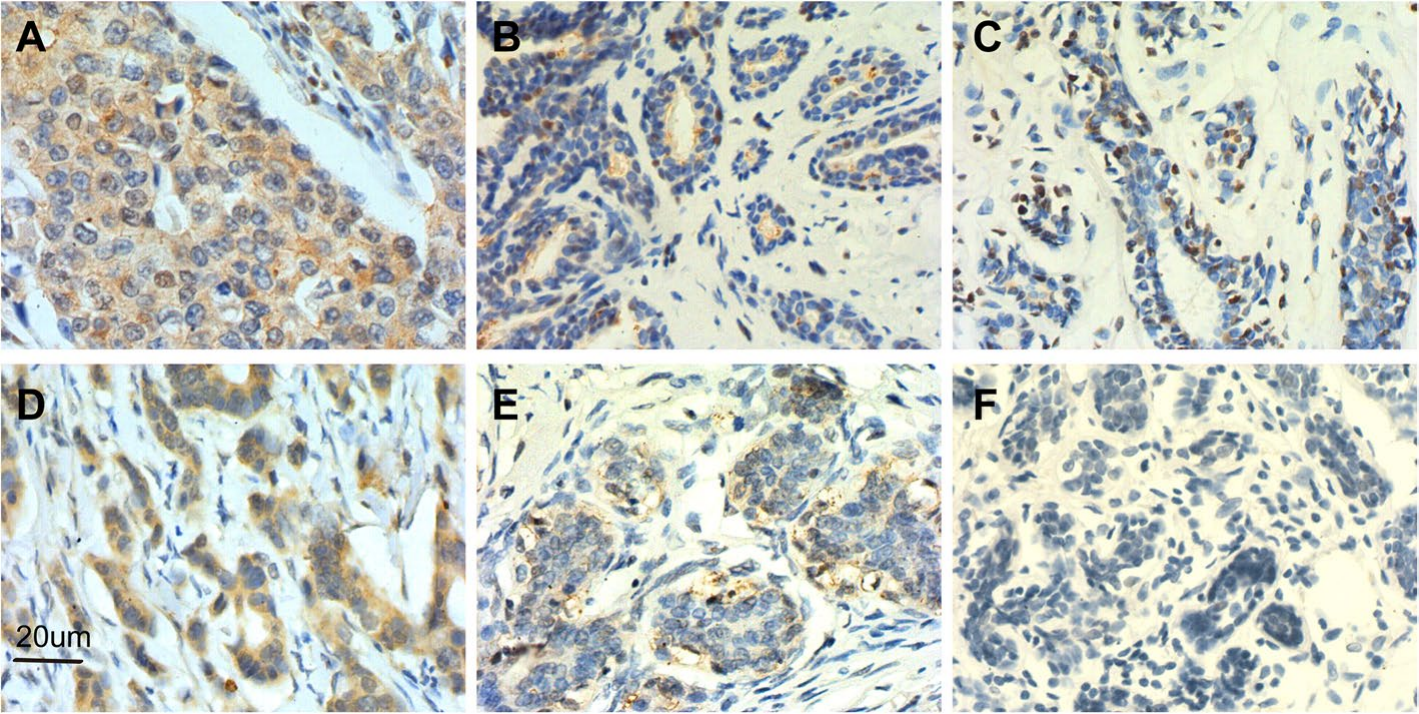
Expression of LEP and LEPR in breast cancer tissues, benign breast tissue and normal para-cancerous breast tissue; Note: High expression of LEP (**A**) and LEPR (**D**) in breast cancer tissues; Low expression of LEP (**B**) and LEPR (**E**) in benign breast tissues; No expression of LEP ( **C**) and LEPR (**F**) in normal para-cancerous breast tissues. Original magnification × 400

LEP and LEPR expression was evaluated in 132 tumors and 66 non-BC patients, which were classified as low expression and high expression. Both LEP and LEPR showed high expression in breast cancer tissues and low expression in benign breast tissues. There was no LEP and LEPR expressed in normal para-cancerous breast tissues (Fig. 2). The proportion of patients with LEP and LEPR high expression in different tissues was shown in Table 2.

**Table 2:** LEP and LEPR expression

Variables	Tumor	Benign lesion	*P*-value	Para-carcinoma tissue	*P*-value
Total number	132	66		30	
LEP High	101	37	0.001*	14	0.003*
LEPR High	93	37	0.005*	17	0.044*

*p*-value were all compared with tumor *LEP* leptin, *LEPR* leptin receptor

The expression of LEP and LEPR were 86.66% (13/15) and 73.33% (11/15) of multiple intraductal papillomas disease, respectively, which resulting high rate of LEP and LEPR in all patients with benign disease. However, the expression decreased to 45.1% and 51.% in patients with benign disease regardless of multiple intraductal papilloma.

The expression of LEP and LEPR did not showed association with menopausal status, histological type, tumor size, tumor grade and metastasis status (*P* > 0.05), as well as the expression of ER, PR (*P* > 0.05). However, the positive rate of LEP expression in patients with lymph node metastasis was 91.8%, which was significantly higher than 67.5% in patients without lymph node metastasis (*P* = 0.002). We also found that LEPR expression was correlated with Ki-67 expression (*P* = 0.002), as shown in Table 3.

**Table 3:** Correlation of clinicopathological features with the LEP and LEPR expression

Clinicopathological features	LEPR expression level (*n* = 132)			LEP expression level (*n* = 132)		
	**High (%)**	**Low (%)**	***P*-value**	**High (%)**	**Low (%)**	***P*-value**
**Menstrual status**			0.820			0.161
Premenopausal	71 (72.4)	27 (27.6)		72 (73.5)	26 (26.5)	
Postmenopausal	24 (70.6)	10 (29.4)		29 (85.3)	5 (14.7)	
**Tumor size**			0.709			0.832
≤ 2 cm	35 (66.0)	18 (34.0)		42 (79.2)	11 (20.8)	
2 ~ 5 cm	52 (73.2)	19 (26.8)		53 (74.6)	18 (25.4)	
> 5 cm	6 (75.0)	2 (25.0)		6 (75)	2 (25)	
**Tumor grade**			0.874			0.397
I	7 (70.0)	3 (30.0)		6 (60.0)	4 (40.0)	
II	43 (72.9)	16 (27.1)		47 (79.7)	12 (20.3)	
III	43 (68.3)	20 (31.7)		48 (76.2)	15 (24.2)	
**TNM stage**			0.849			0.428
0	1 (50.0)	1 (50.0)		1 (50.0)	1 (50.0)	
I	26 (68.4)	12 (31.6)		28 (73.7)	10 (26.3)	
II	49 (70.0)	21 (30.0)		52 (74.3)	18 (25.7)	
III	14 (73.7)	5 (26.3)		17 (89.5)	2 (10.5)	
IV	3 (100.0)	0 (0)		3 (100.0)	0 (0)	
**Histological type**			0.831			0.508
IDC	42 (72.4)	16 (27.6)		45 (77.6)	13 (22.4)	
DCIS	39 (70.9)	16 (29.1)		42 (76.4)	13 (23.6)	
ILC	6 (66.7)	3 (33.3)		8 (88.9)	1 (11.1)	
LIN	6 (60.0)	4 (40.0)		6 (60.0)	4 (40.0)	
**Lymph node metastasis**			0.660			0.002
Yes	36 (73.5)	13 (26.5)		45 (91.8)	4 (8.2)	
No	58 (69.9)	25 (30.1)		56 (67.5)	27 (32.5)	
**Distant metastases**			0.884			0.684
Yes	2 (66.7)	1 (33.3)		2 (66.7)	1 (33.3)	
No	91 (70.5)	38 (29.5)		99 (76.7)	30 (23.3)	
**Estrogen receptor**			0.394			0.564
Positive	71 (72.4)	27 (27.6)		73 (74.5)	25 (25.5)	
Negative	22 (64.7)	12 (35.3)		27 (79.4)	7 (20.6)	
**Progesterone receptor**			0.648			0.298
Positive	68 (69.4)	30 (30.6)		72 (73.5)	26 (26.5)	
Negative	25 (73.5)	9 (26.5)		28 (82.4)	6 (17.6)	
**Ki-67**			0.002			0.487
Positive	75(68.8)	34(31.2)		70(64.2)	39(35.8)	
Negative	8(34.8)	15(65.2)		13(56.5)	10(43.5)	

*LEP* leptin, *LEPR* leptin receptor, *IDC* Invasive Ductal Carcinoma, *DCIS* Ductal Carcinoma In Situ, *ILC* Invasive lobular Carcinoma, *LIN* Lobular Intraepithelial Neoplasia

### LEP and LEPR expression with prognosis

The follow-up time ranged from 22 to 89 months and all patients alive were followed-up for at least 48 months. Eleven patients were lost to follow-up. Two patients refused further treatment after surgery, five were with cancer progression during follow-up, and 7 patients died at last follow-up time. The 5-year overall survival rate of entire cohort was 94.9%.

**Fig. 3:**
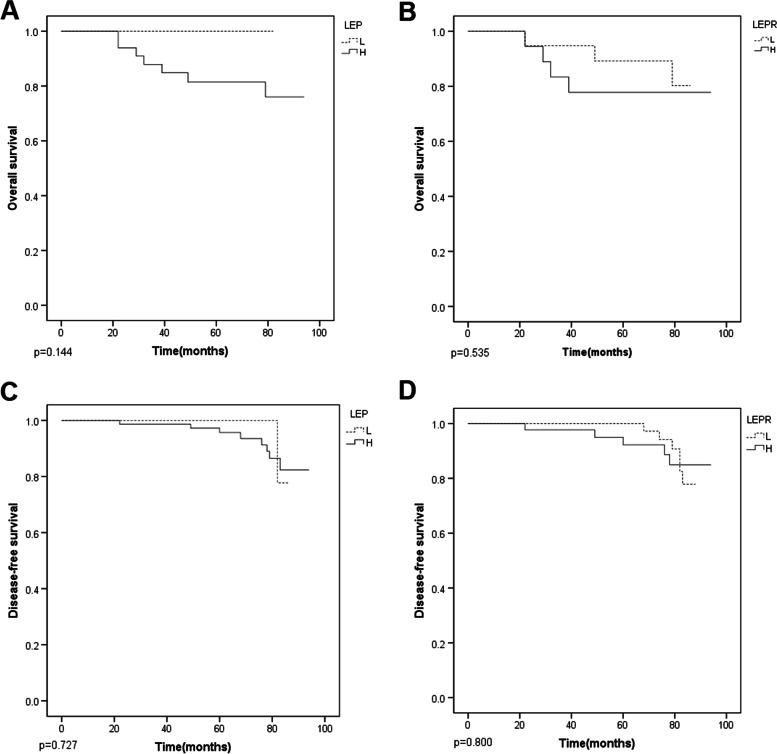
Survival curve of patients with different LEP and LEPR expression. **A**: Overall survival curve comparing patients with high and low expression of LEP; **B**: Overall survival curve comparing patients with high and low expression of LEPR; **C**: Disease-free survival curve comparing patients with high and low expression of LEP; **D**: Disease-free survival curve comparing patients with high and low expression of LEPR

LEP and LEPR expression did not significantly correlated with overall survival, nor with disease-free survival (Fig. 3).

## Discussions

Previous studies have indicated the association between BC and overweight, obesity, excessive nutrition, and metabolic disorders. Incidence of BC was increased with weight gain in middle-aged women [22]. Elevated BMI and WHR were also important risk factors for BC in women [23]. Our previous study also found that BC patients had significantly higher BMI and WHR than patients with benign breast diseases and healthy controls [24]. Kuriyama et al. reported that the increased LEP level and higher BMI would contribute to the increased incidence of BC [25]. Present study partly confirmed previous findings which indicated that LEP/LEPR expression was correlated with progression of BC.

LEPR is a multifunctional single-pass transmembrane protein and widely distributed in many organs [26]. It is the receptor of LEP, which can balance the energy consumption and glucose metabolism by activating JAK2-STAT3 and ERK pathways [27]. Disorder of the processes mentioned above would cause obesity and obesity-related diseases [28]. LEP and LEPR are widely expressed in various tissues including hypothalamus, adipose tissue, nerves, heart, kidney, breast, lung, liver and islet cell surface [29]. Ishikaw et al. detected the expression of LEP and LEPR in 76 cases of invasive ductal carcinoma and 32 cases of normal para-carcinoma breast tissues, and found that the expression of LEPR was significantly higher in tumor epithelium than normal breast epithelium. In addition, LEP was also over-expressed in tumor epithelium than in normal epithelium [30]. Garofalo et al. analyzed 148 BC tissue and benign breast lesions indicating that LEPR was expressed in 41.2% of BC tissues but not in benign breast lesions [31]. Previous study analyzed the expression of LEP, long isoform of LEPR and short isoform of LEPR in 322 primary BC tissues and found that the long isoform of LEPR and the short isoform of LEPR were expressed in all tumor tissues and LEP was expressed in 318 samples [32]. In addition, the expression of these three proteins was positively associated with expression of estradiol and progesterone receptors, but not with tumor diameter and malignant degree. Present study showed that LEPR was expressed in 70.5% of tumor tissues, which was significantly higher than 56.3% of benign breast tissues and 44.0% of normal para-carcinoma tissues. A previous study of our team showed that LEP was over-expressed in BC tissues and significantly associated with LEPR expression [33]. The co-expression of LEP and LEPR in primary BC showed that LEP expressed on mammary tumor cells via an autocrine pathway [18]. Highly expressed LEPR in BC make them more sensitive to LEP stimulation. LEP is associated with tumor cell migration and invasion, as well as angiogenesis in some tumors. It is also involved in several signaling pathways, as JAK/STAT, protein kinase B, phosphatidylinositol 3-kinase and mitogen activated protein kinase. These pathways, controlled by LEP-LEPR, are strongly related to cell survival and differentiation [34, 35].

LEP expression might be correlated with lymph nodes metastases of BC. Garofalo et al. found that LEPR expression rate was 51.5% in BC tissues of patients with lymph node metastasis, which was significantly higher than 41.2% in BC tissues of patients without lymph node metastasis [33]. In contrast, another study showed that low LEPR expression increased the risk of lymph node metastasis by fourfold [36]. Present study found that LEPR expression did not significantly correlated with lymph node metastases (*p* = 0.66), but the expression rate of LEP in patients with lymph node metastasis was significantly higher than that of patients without lymph node metastasis (*p* = 0.002). It indicated that LEP is involved in the proliferation process of BC and plays a very important role in the development of BC. Moreover, this study also found that the expression of LEP and LEPR were balanced when stratified by patients’ age, menopausal status, tumor size, tumor pathological classification, distant metastasis and the expression of ER and PR, which was consistent with previous study [30, 37].

Another finding of present study was that LEPR expression was correlated with Ki-67 (*P* = 0.002). Ki67 was related to cell proliferation cycle and its expression has been correlated with the development of a variety of malignant tumors. The results suggested that LEPR could promote BC cell proliferation after activation.

Previous study has shown an association between high LEP expression levels and poor prognosis in several cancers [38]. Other reported low LEP expression and high expression of Ob-R mRNA in breast cancer tissue correlated with shorter OS and RFS [39]. LEP and LEPR expression did not correlated with overall survival, nor disease-free survival in our analysis. Similar results have been reported in oral and oropharyngeal cancer [36]. However, another study found that the expression of LEP was significantly associated with overall survival. Further more, for the postmenopausal patients or triple negative patients and lymph node metastasis patients, LEP-positive group had worse prognosis [40].

There were also several limitations of present study. First, there was unavoidable biases due to the retrospective nature of present study. Second, this was a study with small sample size which resulting in limited reliability. Thus, all conclusions should be interpreted cautiously.

## Conclusion

In conclusion, the positive rate of LEP and LEPR expression in BC tissues was significantly higher than that in benign breast tissues and normal para-carcinoma tissues. The LEP and LEPR expression were significantly correlated with lymph node metastasis and Ki-67 expression, respectively. High LEP/LEPR expression were risk factors for occurrence of BC, but without impact on survival.

## Data Availability

The datasets generated and analyzed during the current study are available from the corresponding author on reasonable request.

## Abbreviations

BC: Breast cancer
BMI: Body mass index
DAB: Diaminobenzidine
ER: Estrogen receptor
LEP: Leptin
LEPR: Leptin receptor
PR: Progesterone receptor
SD: Standard deviation
WHR: Waist to hip
